# Supplementary material for: Genome-Wide Analysis of Genes Involved in the GA Signal Transduction Pathway in ‘duli’ Pear (Pyrus betulifolia Bunge)
Source: Int J Mol Sci. 2022 Jun 12;23(12):6570. doi: 10.3390/ijms23126570 (PMC9224306; doi:10.3390/ijms23126570)
Supplement: Supplementary file 1 [file ijms-23-06570-s001.zip › Figures S1-S3.pptx]

## Slide 1
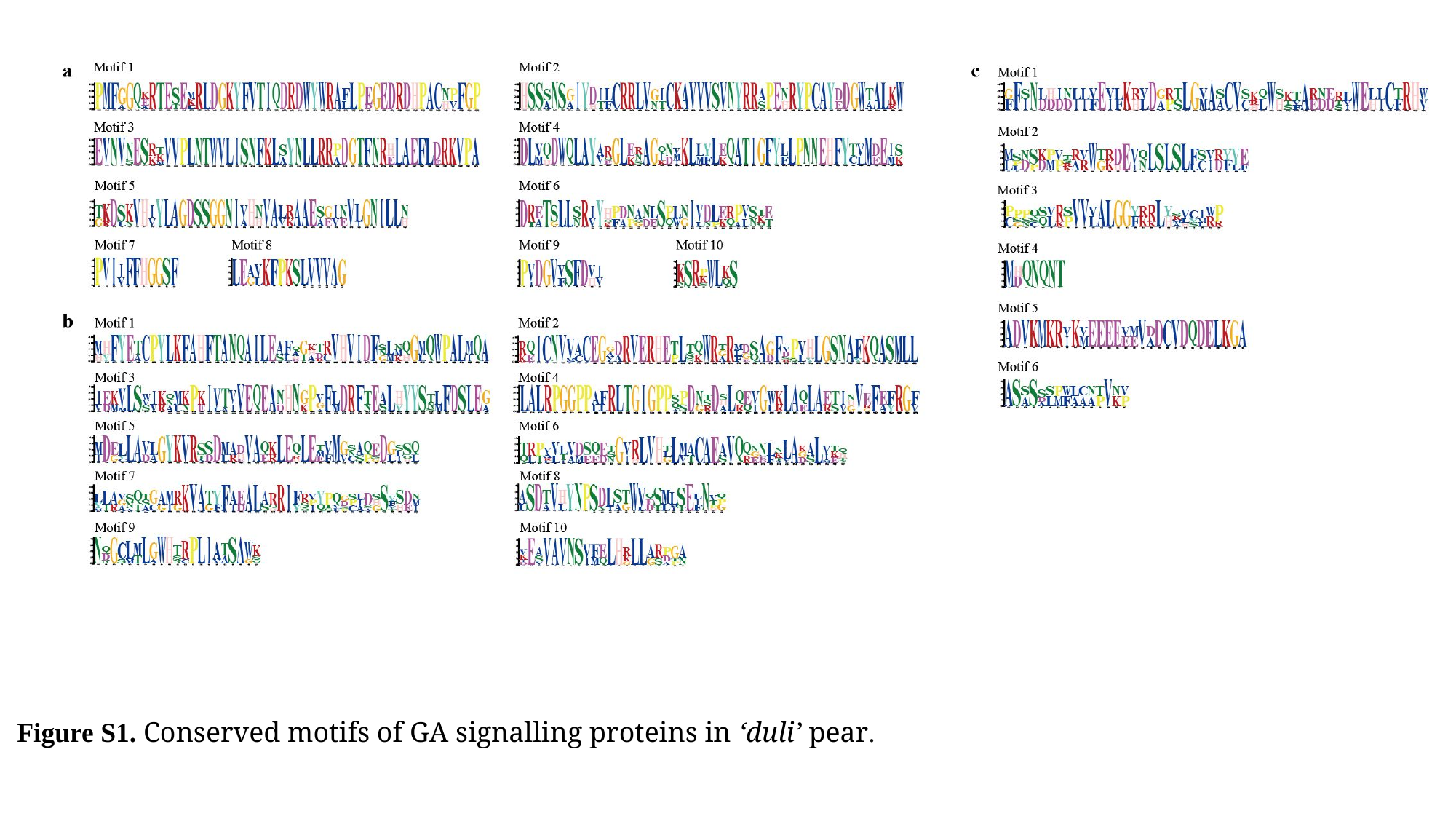

Figure S1. Conserved motifs of GA signalling proteins in ‘duli’ pear.

## Slide 2
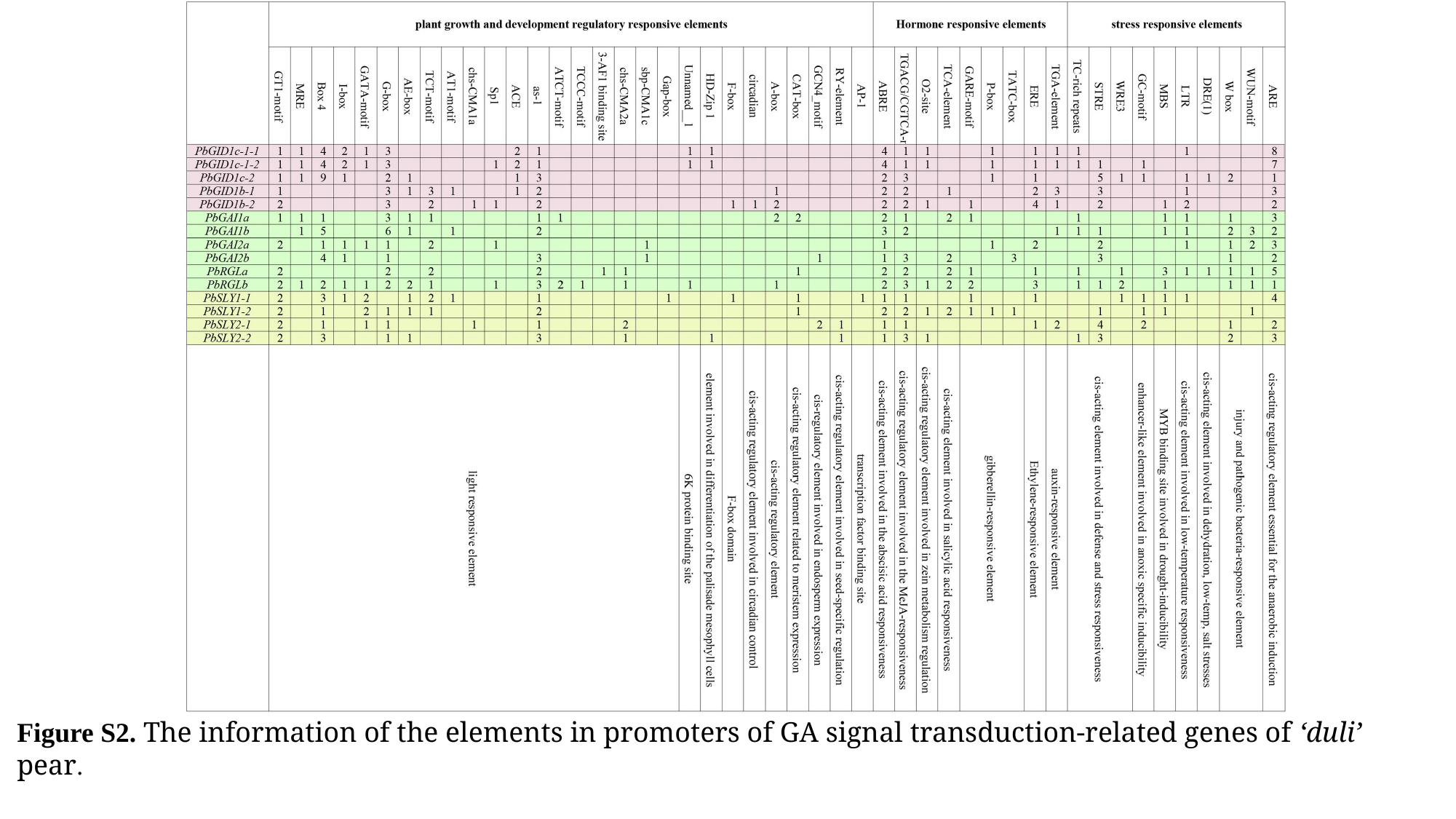

Figure S2. The information of the elements in promoters of GA signal transduction-related genes of ‘duli’ pear.

## Slide 3
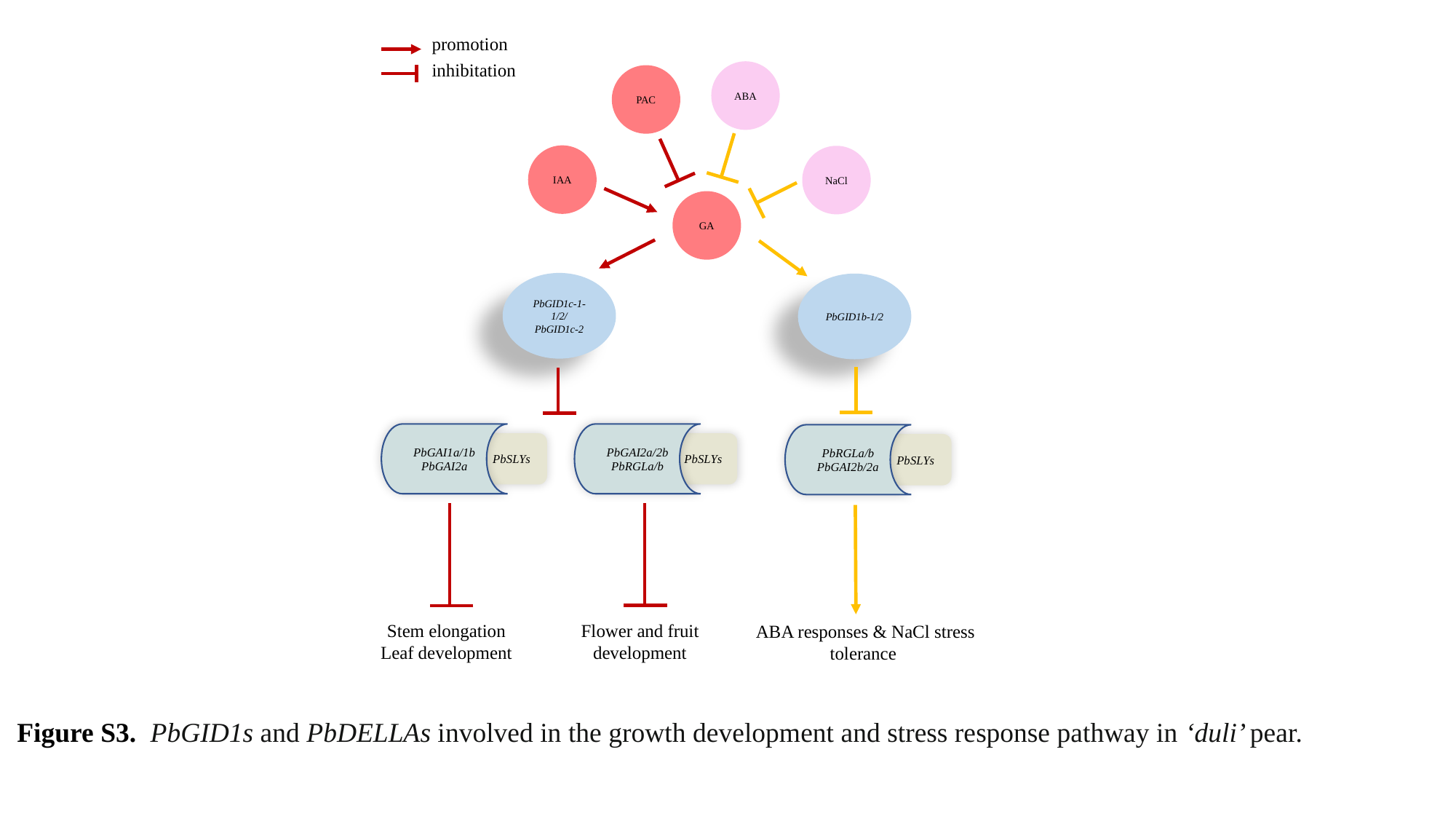

promotion
inhibitation
ABA
PAC
IAA
NaCl
GA
PbGID1c-1-1/2/PbGID1c-2
PbGID1b-1/2
PbGAI1a/1b
PbGAI2a
PbGAI2a/2b
PbRGLa/b
PbSLYs
PbRGLa/b
PbGAI2b/2a
PbSLYs
PbSLYs
Stem elongation
Leaf development
Flower and fruit development
ABA responses & NaCl stress tolerance
Figure S3. PbGID1s and PbDELLAs involved in the growth development and stress response pathway in ‘duli’ pear.
