# Supplementary material for: Genome-Wide Analysis of Genes Involved in the GA Signal Transduction Pathway in ‘duli’ Pear (Pyrus betulifolia Bunge)
Source: Int J Mol Sci. 2022 Jun 12;23(12):6570. doi: 10.3390/ijms23126570 (PMC9224306; doi:10.3390/ijms23126570)
Supplement: Supplementary file 1 [file ijms-23-06570-s001.zip › Table S1.pdf]

Table S1. Ka/Ks values of genes in *GID1*, *DELLA* and *SLY* families of '*duli*' pear (*Pyrus betulifolia* Bunge) and *Arabidopsis thaliana*

Gene Name	Gene name	Ka	Ks	Ka/Ks
PbGID1c-1-1	*AtGID1a*	0.157225719	3.438639615	0.045723233
PbGID1c-1-2	*AtGID1a*	0.155543594	3.606557397	0.043127996
PbGID1c-2	*AtGID1a*	0.168608174	2.334620841	0.072220795
PbGID1c-2	*AtGID1c*	0.153344137	2.428625109	0.063140308
PbGID1b-2	*AtGID1b*	0.150773442	2.21429697	0.068090886
PbGID1b-1	*AtGID1b*	0.145015836	2.320899644	0.062482596
PbGAI2b	*AtRGL3*	0.290507113	2.929148544	0.099178006
PbGAI2a	*AtRGL2*	0.276715671	3.817954832	0.072477461
PbGAI2a	*AtRGL3*	0.30038569	2.529252459	0.118764613
PbGAI1b	*AtGAI*	0.19406061	2.272881475	0.085380907
PbGAI1a	*AtGAI*	0.196948284	2.408199203	0.081782389
PbSLY2-1	*AtSLY2*	0.319643429	1.835456686	0.174149263
PbSLY2-2	*AtSLY2*	0.384011923	2.561380002	0.149923839
PbSLY1-2	*AtSLY1*	0.241703517	2.686362608	0.089974271
