# Supplementary material for: Genome-Wide Analysis of Genes Involved in the GA Signal Transduction Pathway in ‘duli’ Pear (Pyrus betulifolia Bunge)
Source: Int J Mol Sci. 2022 Jun 12;23(12):6570. doi: 10.3390/ijms23126570 (PMC9224306; doi:10.3390/ijms23126570)
Supplement: Supplementary file 1 [file ijms-23-06570-s001.zip › Table S2.pdf]

Table S2. The sequences of primers used for qRT-PCR

Gene Name	Forward Primer (5'-3')	Reverse Primer (5'-3')
PbACTIN	TTGGTATGGGTCAGAGG	CTGTGAGCAGAACTGGGTG
PbGID1c-1-1	ATGTGTTGGGTTTTCTTCAAG	CGGCGGCACAGAATA
PbGID1c-1-2	GTAAATGAATCCAGGACAGT	CGGCGGCACAGAATA
PbGID1c-2	CCTTCAACCGCCACT	ATGTTCCCGCCAGAG
PbGID1b-1	CGCCATCTACGACACCT	TGCCATTCCCGCTCT
PbGID1b-2	ATCGGTATCCTTGTGCTTAT	CTGCAAACATTGGGTGG
PbGAI1a	TCCCTCCGAACTCCTCTT	GTAGCCGATCTGGGTCATC
PbGAI1b	GCGAACAGCCAGGATAAGG	CCAGCCCAACATCAGACAC
PbGAI2a	CCAGCGGACTCGGGTTT	GGTAAGGGCAGGTCTCGTAA
PbGAI2b	CCCAACTGGCCGAAACTA	TGCCTGCCCAAATACACC
PbRGLa	GCCGCAGCAGAACCACA	GCCACGCTGGACGGATT
PbRGLb	CGAAACATGGACCGACAAC	GCAAGGGCCTGAATAAGC
PbSLY1-1	CGGATGTAAAGATGAAGAGGG	CGCCGATGAGAAAGAAGG
PbSLY2-1	CACCGTCGCCAGAAATG	CCTGTAGAGCCTCTTGTAGCC
PbSLY1-2	GATGGCAGCGGCGAGTT	CGACGAAGAAGAAGGAGCAGA
PbSLY2-2	CGGCGGCTACAAGAAG	AACGACGACGGGTTTCA
